# Diagnosis and management of coronavirus disease-associated immune thrombocytopenia: a case series

**DOI:** 10.1590/0037-8682-0029-2021

**Published:** 2021-04-28

**Authors:** Fatma Yılmaz Aydın, Vehbi Demircan

**Affiliations:** 1 University of Dicle, School of Medicine, Department of Internal Medicine, Diyarbakır, Turkey.; 2 University of Dicle, School of Medicine, Department of Hematology, Diyarbakır, Turkey.

**Keywords:** Immune thrombocytopenia, Thrombocytopenia, Coronavirus disease

## Abstract

Data on health problems and fatal complications associated with coronavirus disease (COVID-19) have consistently been reported. Although immune thrombocytopenia has been associated with multiple viral infections, only few studies have shown its association with COVID-19. Here, we have reported a case series of two cases pertaining to patients diagnosed with COVID-19-associated immune thrombocytopenia, elaborating on the clinical course, management, and response to treatment.

## INTRODUCTION

Immune thrombocytopenia (ITP) is an uncommon disease with a platelet count of <100 × 10^3^/uL, mostly with minor hemorrhages^1^. In this disease, isolated thrombocytopenia develops due to de novo or autoantibody-mediated destruction of platelets secondary to an underlying disorder. The causes of secondary ITP include autoimmune, lymphoproliferative, and collagen vascular diseases^2^. ITP is also associated with many infections, most of which are virus induced. Hepatitis C virus, human immunodeficiency virus, herpes viruses, cytomegalovirus, Epstein-Barr virus, parvovirus, measles, and rubella are common viral causes of ITP^2^.

Coronavirus disease (COVID-19), caused by severe acute respiratory syndrome coronavirus 2, which was first reported in December 2019, is a serious infection that has caused a global pandemic^2^. Data on various health problems and fatal complications caused by COVID-19 have been reported in the literature. Sepsis, in which the inflammatory response plays a role in pathogenesis, increases mortality^3^. Hematological problems including lymphopenia, thrombocytopenia, and disseminated intravascular coagulation***(*** DIC) are common hematological conditions associated with COVID-19. Thrombocytopenia is a serious risk factor for increased morbidity and mortality, especially in severe COVID-19 cases^4^. However, the link between ITP and COVID-19 has rarely been reported in the literature^4^ ^,^ ^5^. Here, we have presented a case series of two cases pertaining to patients with COVID-19-associated ITP.

## CASE REPORT

**FIGURE 1:**
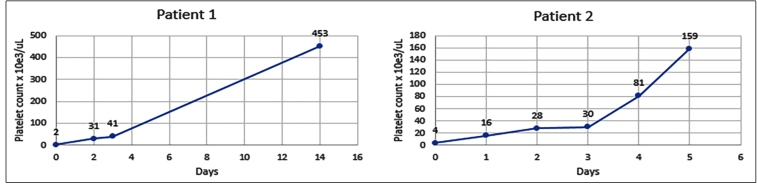
The course of platelet counts in two patients with COVID-19-associated ITP.

The first patient was a 42-year-old woman who presented with nasal bleeding and widespread petechiae and purpura on the skin. She had been diagnosed with epilepsy before 4 years. Three weeks before admission, the patient developed diarrhea and vomiting and was then diagnosed with COVID-19 on a polymerase chain reaction (PCR) test. She was administered hydroxychloroquine treatment for 5 days. Additionally, thorax computed tomography (CT) revealed bilateral ground-glass opacities. She was admitted to our hospital after multiple nosebleeds and petechiae purpura on the 21st day. A laboratory blood test revealed a thrombocyte count of 2 × 10^3^/uL (Table 1). Peripheral blood smear showed no schistocytes. After excluding DIC, microangiopathic diseases (such as thrombotic thrombocytopenia purpura and hemolytic uremic syndrome), other infections, drugs, and antiphospholipid antibodies that might have caused thrombocytopenia, the patient was diagnosed with COVID-19-associated ITP. To control the severe thrombocytopenia, two units of platelet transfusion were administered. Treatment with prednisolone (1 mg/kg/day) and intravenous immunoglobulin (IVIG; 1 g/kg) was initiated. After 2 days of prednisolone and IVIG therapy, her platelet count increased to 41 × 10^3^/uL. Since the patient’s platelet count was 453 × 10^3^/uL and prednisolone treatment was continued for 1 week, the dose was slowly tapered and discontinued (Figure 1).

**TABLE 1:** Characteristics of the two patients with COVID-19-associated ITP.

	Patient 1	Patient 2	Reference range
Age (years)	42	33	
Sex	Female	Female	
Hemoglobin level (g/dL)	9.7	13.5	12.9-14.2
Platelet count (× 10^3^/uL)	2	4	155-366
Leucocyte count (× 10^3^/uL)	10.2	7.19	3.7-10.1
Lymphocyte count (× 10^3^/uL)	0.58	0.67	1.09-2.99
PT (s)	12.5	12	10-14
APTT (s)	22.2	23.1	21-29
D-dimer (mg/L)	0.4	0.38	0.08-583
Lupus anticoagulant	Negative	Negative	
Anti-cardiolipin antibodies	Negative	Negative	
Anti-nuclear antibodies	Positive	Negative	
Virus serology			
Human immunodeficiency virus	Negative	Negative	
Hepatitis B and C	Negative	Negative	
Epstein-Barr virus	Negative	Negative	
Parvo B19 virus	Negative	Negative	
Cytomegalovirus virus	Negative	Negative	
HSV virus	Negative	Negative	

The second patient was a 33-year-old woman with a history of chronic hepatitis B infection. She presented with cough and fever. Subsequently, she was diagnosed with COVID-19 on a PCR test and was administered favipiravir treatment. The patient presented with petechiae, purpura, and severe vaginal bleeding on the four day after diagnosed with COVID-19. A laboratory test for complete blood count revealed a platelet count of 4 × 10^3^/uL (Table 1), while the peripheral smear revealed normal findings, except for isolated thrombocytopenia. The patient underwent two units of apheresis platelet transfusion. Corticosteroid administration was considered, but not initiated due to insufficient and limited data at that time and the concern that it may worsen outcomes during the active replicative phase of COVID-19. Only two courses of IVIG (1 g/kg) were administered. On the second day after initiation of IVIG, her platelet count increased to 28 × 10^3^/uL. The patient was discharged after the platelet count was 159 × 10^3^/uL on the 5th day of the treatment (Figure 1). Based on the exclusion of other causes of ITP and the rapid improvement in platelet count with treatment, we diagnosed the patient with COVID-19-associated ITP.

## DISCUSSION

The exact mechanism of ITP is not fully understood. Many researcher have suggested that viral infection triggers the disease and that pre-formed antibodies cross-react with platelet surface integrins such as glycoprotein Ib-IX-V or glycoprotein IIb/IIIa^6^ ^,^ ^7^. Although ITP has been associated with many viral infections^8^, there are limited data on its association with COVID-19. The diagnosis of COVID-19-associated ITP may be difficult due to several other potential causes, such as coagulation activation by COVID-19, leading to DIC and subsequent thrombocytopenia. Zulfiqar et al. have reported a case of COVID-19-associated ITP, which developed during the active phase of the infection^5^. Contrastingly, in a case series of three cases, Bomhof et al. found that ITP can develop not only during active infection but also after resolution of COVID-19 symptoms^4^. Thrombocytopenia developed in one of our patients approximately 2 weeks after recovery of COVID-19 symptoms, while the other patient developed ITP in the severe symptomatic period. Therefore, ITP can occur not only during active infection but also after many weeks of alleviation of COVID-19 symptoms.

The purpose of ITP therapy is to prevent severe bleeding by providing adequate platelet. ITP treatments include glucocorticoids, IVIG, or thrombopoietin receptor agonists^9^. IVIG is usually preferred in cases where a rapid increase in platelet count is required. Since IVIG inhibits the phagocytosis of macrophages, treatment with IVIG may be successful in the early stages of COVID-19^8^. We preferred IVIG treatment in our patients primarily because it arrested severe thrombocytopenia and active bleeding. Glucocorticoids, another treatment option, are the primary management modality for ITP^9^. In our first patient, we used prednisolone treatment for 2 weeks in addition to IVIG treatment since thrombocytopenia developed after COVID-19 symptoms improved and the PCR test revealed negative results. We did not prefer thrombopoietin receptor agonists in our patients due to the high risk of thromboembolism in patients with severe COVID-19. Previously, corticosteroid administration was not recommended by the World Health Organization (WHO) for the treatment COVID-19^10^. Historically, the use of corticosteroids during the severe acute respiratory syndrome and Middle East respiratory syndrome epidemics has been known to suppress the immune response^11^. Since then, evidence supporting benefits of corticosteroid treatment for some patients with COVID-19 has been increasing^12^.

Early diagnosis and treatment of this complication of COVID-19 can lead to better outcomes. Clinicians should adopt an individualized approach to evaluate the risks and benefits when initiating ITP treatments.
